# Birth defects in a cohort of infants born to HIV-infected women in Spain, 2000-2009

**DOI:** 10.1186/s12879-014-0700-3

**Published:** 2014-12-24

**Authors:** Luis M Prieto, María Isabel González- Tomé, Eloy Muñoz, María Fernández-Ibieta, Beatriz Soto, Ana Álvarez, Maria Luisa Navarro, Miguel Ángel Roa, José Beceiro, María Isabel de José, Iciar Olabarrieta, David Lora, José Tomás Ramos, the Madrid Cohort of HIV-Infected Mother-Infant Pairs

**Affiliations:** Department of Paediatrics, Hospital Universitario de Getafe, Madrid, Spain; Department of Immunodeficiencies, Hospital Universitario 12 de Octubre, Madrid, Spain; Department of Gynecology, Hospital Universitario 12 de Octubre, Madrid, Spain; Department of Pediatric Infectious Diseases, Hospital General Universitario Gregorio Marañón, Madrid, Spain; Department of Paediatrics, Hospital General de Móstoles, Madrid, Spain; Department of Paediatrics, Hospital Principe de Asturias, Madrid, Spain; Department of Pediatric Infectious Diseases, Hospital Universitario La Paz, Madrid, Spain; Department of Paediatrics, Hospital Severo Ochoa, Madrid, Spain; Department of Epidemiology, Hospital Universitario 12 de Octubre, Madrid, Spain

**Keywords:** Antiretrovirals, Birth defects, HIV

## Abstract

**Background:**

Antiretroviral therapy (ART) in pregnancy has resulted in a marked impact on reducing the risk of mother-to-child transmission (MCT) of HIV. However the safety of in utero ART exposure in newborns remains a concern.

**Methods:**

A multicenter prospective observational study of HIV-infected mother and their infants was performed in Madrid, Spain, from 2000 to 2009. Children had regular visits with clinical examination according to protocol until the age of 24 months. An abdominal ultrasound and an echocardiogram were scheduled during follow up. Birth defects (BDs) were registered according to European Surveillance of Congenital Anomalies (EUROCAT).

**Results:**

A total of 897 live births from 872 mothers were included. Overall the birth defects prevalence observed was 6.9% (95% CI 5.4-9.1).The most commonly reported birth defects types were in genital organs and urinary system (19 cases, 30.6%) and cardiovascular system (17 cases, 27.4%). There was no increased risk for infants exposed in the first trimester to ARVs compared with unexposed infants. No significant associations were observed between exposure to any individual antiretroviral agent during pregnancy and birth defects

**Conclusion:**

A higher prevalence of BDs was observed, higher than previously reported. In utero exposure to ART was not proved to be a major risk factor of birth defects in infants. However the relatively small number of patients is a major limitation of this study.

## Background

Antiretroviral therapy (ART) in pregnancy has a marked impact on reducing the risk of mother-to-child transmission (MCT) of HIV [1]. In 1994, the use of zidovudine (ZDV) given during pregnancy and delivery to the mother and during the first weeks of life to the nonbreast-fed newborn was demonstrated to reduce transmission by about 67% [2]. It is now recommended worldwide that HIV-infected pregnant women receive combination ARV regimens during pregnancy [3]. An increasing number of pregnant women are receiving ART in Spain [4],[5]. The use of HAART during pregnancy in Spain has reduced the perinatal HIV transmission below 2% [5].

There are concerns about potential adverse events in newborns exposed to ART during pregnancy, including birth defects. Different prevalences of birth defects (BDs) have been reported in studies with large number of subjects from Europe and United States. While some studies have not detected an overall increase in the prevalences of birth defects (BDs) associated with ART exposure in pregnancy [6],[7], others studies have shown an increased prevalence [8],[9].

Some studies have reported elevated risks with specific exposures. Nucleoside reverse transcriptase inhibitors have a high transplacentary passage [10]-[12]. These drugs have been associated with mitochondrial DNA depletion and, in preliminary studies in monkeys, mitochondrial toxicity has been found in the skeletal and cardiac muscles and brain tissue [10],[12]. A significantly increased prevalence of hypospadias following first trimester zidovudine exposure, compared with later on or no exposure has been observed [13]. Recently, a specific association between in utero exposure to zidovudine and heart defects have been reported in the French Perinatal Cohort [14]. Likewise an increased BDs prevalence in infants exposed to didanosine in first trimester was reported by the Antiretroviral Pregnancy Registry [6].

Exposure to efavirenz, a non-nucleoside reverse transcriptase inhibitor (NNRTI) has been associated with central nervous system defects in monkeys [15], and case reports of neural tube defects in human patients are concerning [16],[17]. A recent meta-analysis has not shown increased risk of birth defects, including central nervous system defects, in newborns exposed to efavirenz during the first trimester of pregnancy (RR 0.85 (95% CI 0.61-1.20) [18]. This result was an important consideration by WHO recommendation for the use of efavirenz during pregnancy [19]. However new data about central nervous system defects published, beyond this meta-analysis, has been less reassuring in the consideration of the use of EFV during first trimestrer of pregnancy [14].

The continued surveillance of birth defects in newborns exposed to antiretrovirals in utero is required, more even when newer antiretroviral agents become available. The aim of our study was to estimate the prevalence of birth defects in children exposed in utero to ARV drugs and to assess the association between in utero exposure to antirretovirals and birth defects in the Madrid Cohort of HIV-infected mother-infants pairs.

## Methods

### Study population

The Madrid Cohort of HIV-infected mother-infants pairs is a multicenter prospective observational study of HIV-infected pregnant women and their infants followed up since birth. Beginning in 2000, pregnant women and infants were recruited in 8 hospitals in Madrid, Spain. All participants gave verbal informed consent and the study was approved by the Clinical Research Ethics Committee at Hospital Universitario de Getafe.

The baseline characteristics of the cohort and the mother-to-child transmission of HIV data have been provided previously [5].A collaboration of infectious disease specialists, gynaecologists and paediatricians provided prospective and active follow-up as soon as pregnancy was detected in previously monitored HIV-positive women, or as soon as a pregnant woman was identified as HIV-positive, in order to determine the mother-to-child transmission rate, the effectiveness and safety of ART, the presence of birth defects and age-appropriate development in children follow-up.

The cohort for the current analyses includes pregnancies with a definitive outcome through December 31, 2009.

### Characteristics of the study population and variables

Women were visited during pregnancy, at delivery and at postpartum. Women with a previous diagnosis of HIV infection were enrolled in the first visit of pregnancy. Women who were diagnosed with HIV infection during pregnancy were enrolled in the first visit after the diagnosis. Information on maternal demographic characteristics was recorded. At each visit, a medical history and a physical examination was performed. Blood count, biochemistry panel, HIV-1 viral load (plasma quantitative HIV-1 RNA level) and CD4^+^ lymphocyte counts and percentage were collected at each scheduled visit. Start and stop dates were recorded for each antiretroviral agent used. Maternal viral load less than 50 copies/ml was defined as ‘undetectable’.

Trimesters of pregnancy were defined as follows: first trimester, the first day of the last menstrual period through 13 completed weeks of gestation; second trimester, 14 through 28 completed weeks of gestation; and third trimester, beginning at 29 weeks of gestation until delivery.

ART in pregnant women was classified as: none, monotherapy with zidovudine, dual therapy and any combination of 3 or more drugs was categorized as HAART. HAART was further categorized according to whether a protease inhibitor (PI) or a nonnucleoside reverse transcriptase inhibitor (NNRTI) was included. ART treatment administered during pregnancy was recorded, with the dates when started and stopped. Exposure status for each drug was categorised as unexposed, exposed during first trimester, or exposed since the second or third trimester. Birth defects were studied associated to exposure to each drug in first or since second or third trimester compared to no exposure.

A self-report of illicit drug use or a positive urine test result for heroin or opiates, methadone or cocaine was registered. Other variables like cigarette smoking and alcohol use were also self-reported. Diabetes was included as a maternal condition in the analysis. However no other drugs different from ARV were analysed.

Stillbirths and termination of pregnancy are not considered in this analysis. Newborn/infant visits occurred at birth, after 2 or 3 and 6 weeks and thereafter 3, 6, 12, 18 and 24 months. Children had regular visits with clinical examination according to protocol until the age of 24 months. Each visit included a medical history and a physical examination. An abdominal ultrasound was also performed in the first month of life, and an echocardiogram during the first 24 months of life by protocol.

‘Premature birth’ was defined as children born before 37 weeks of pregnancy. ‘Low birth weight’ was defined as children born weighing from 1.500 to 2.500 g; ‘very low birth weight’ was defined as 1.499 g or less.

### Birth defects

Birth defect was defined as a structural defect of the body or organ system that may affect viability, quality of life and often requiring surgery [20].

Abnormalities in the fetus/newborn/infant were determined from all available data, including prenatal ultrasound examinations, physical examinations in the newborn period and at each follow-up visit.

Abnormalities observed in the active surveillance system including abdominal ultrasound in neonatal period or echocardiograms during follow-up, as scheduled in the follow-up of this cohort, were also included.

BDs were recorded and classified according to EUROCAT (European Surveillance of Congenital Anomalies) [21], a simpler system than that proposed by the Antiretroviral Pregnancy Registry (APR) [22]. A baby with multiple defects was counted as a single outcome. Birth defects were grouped according to an organ system classification: central nervous system, eyes, face (including neck and ears), hepatic/digestive, musculoskeletal (including cranial bone alterations), cardiac, genitourinary, chromosomal, extremities and others [23].

“Minor” anomalies are excluded, when isolated, because they have lesser medical, functional or cosmetic consequences. We also exclude anomalies which are not always truly congenital in origin, sometimes associated with immaturity at birth.

Angiomas, congenital nevi, pre-auricular appendices, sinus nodules, sacral dimples, atresia of the tears ducts, ovarian cysts, hydrocele, hip dislocation not associated to dysplasia, renal pelvis dilatation of less than 10 mm (without hydronephrosis or urethral atresia), inguinal and umbilical hernias were not classified as BDs and therefore also not included.

These cases have not been reported previously to the Antiretroviral Pregnancy Registry or any other regulatory authorithy.

### Statistical analysis

The categorical variables were expressed as frequencies and percentages, while numerical variables were presented as means, medians, standard deviation, and interquartile range. The categorical variables were compared using the chi square, linear tendency chi square or Fisher’s exact test when considered appropriate. Continuous variables were analyzed with ANOVA test, or, in case of non normal distributions, using a non-parametric test. Regression logistic model was used to estimate the strength of association between birth defect and ARV trimester exposure. Results are presented as crude and adjusted odds ratio and 95% confidence intervals (CIs) adjusted for alcohol and illegal drug use during pregnancy. All p values are two-sided p-values and a p value of < 0.05 was considered as statistically significant. The data were analysed with the SPSS (Chicago, IL) program, version 15.0 for Windows.

## Results

### Maternal and infants characteristics and antiretroviral therapy

During the study period 898 children, including 25 twins or triplets, were considered for this analysis.

The baseline characteristics of the mothers by antiretroviral exposure (exposure in first trimester, exposure in second/third trimester, no antiretroviral exposure in pregnancy) are compared in Table 1. The median gestational age at inclusion was 12 weeks (interquartile range 8–19 months). Women receiving ART during first trimester were more likely to be white and older. Women without antiretroviral therapy were more likely to use illicit drugs or to drink alcohol.

**Table 1:** **Baseline characteristics of HIV infected mothers and time of earliest antiretroviral therapy during pregnancy**

Characteristics	First trimester (n = 329)	Second/third trimester (n = 488)	No antiretroviral treatment (n = 80)	***P***
Mean age (y) (n = 825)	33.3	30.3	29.8	<0.001
Race (n = 898)				< 0.001
Caucasian	279 (84.8%)	307(62.9%)	41 (51.2%)	
Sub-Saharan	33 (10.0%)	95 (19.5%)	27 (33.7%)	
Latino American	13 (4.0%)	74 (15.2%)	9 (11.2%)	
Others	4 (1.2%)	12 (2.4%)	3 (3.8%)	
Smoking during pregnancy	123 (41.8%)	136 (30.8%)	11 (16.1%)	0.05
Alcohol use during pregnancy (n = 696)	10 (3.6%)	16 (4.4%)	8 (16.3%)	< 0.001
Illegal drug use during pregnancy (n = 750)	22 (7.4%)	35 (8.9%)	17 (27.9%)	< 0.001
CD4+ lymphocyte count at enrolment (n = 554)				0.61
>500 cells/μl	121 (48.2%)	123 (42.7%)	6 (40%)	
200-500 cells/μl	103 (41.0%)	127 (44.1%)	8 (53.3%)	
<200 cells/μl	27 (10.8%)	38 (13.2%)	1 (6.7%)	
HIV RNA levels to delivery (n = 642)				<0,001
<50 copies /ml	224 (81.4%)	245 (70.6%)		
>50 copies/ml	51 (18.6%)	102 (29.4%)	20 (100%)	

Women had a good immunological condition status at baseline, and there was no significant difference observed in CD4 lymphocyte counts between the three groups.

Eighty women (8.9%) did not receive treatment during pregnancy. For those who treatment was known during pregnancy, ten women (1.2%) received monotherapy with zidovudine; 21 women (2,6%) received dual therapy (zidovudine plus lamivudine or nevirapine) and 709 (86.6%) received HAART. More than a third of infants (36.7%) had early in-utero exposure.

Four hundred and seventy one infants (52.9%) were males. The median gestational age was 38 completed weeks [interquartile range (IQR): 37–39 weeks]. One hundred and ninety four infants were premature (22.8%); 221 infants (25.6%) presented low weight at birth. The median time of follow-up of the children was 18 months (interquartile range 12–24 months).

### Birth defects

Among the study population, major birth defects were recorded in 62 infants, presenting an overall prevalence of 6.9% (95% CI: 5.3-8.7). There were 70 anomalies identified in these 62 infants. Minor defects, classified according EUROCAT, were excluded.

The prevalence of birth defects among HIV-infected women with first-trimester antiretroviral exposure was 7.0% (95% CI: 4.5-10.3). No significant differences were found in the BD prevalence of children receiving intrauterine ART during the first trimester of pregnancy compared with those who started later [7.0% (23/329) vs 6.8% (39/568); p = 1.00] or those non exposed to antiretroviral treatment during pregnancy [7.0% (23/329) vs 7.5% (6/80); p = 0.80].

The most commonly reported birth defects were in genital organs and urinary system (19 cases, 30.6%), cardiovascular system (17 cases, 27.4%), musculoskeletal (8 cases, 12.9%) and digestive system (6 cases, 9.7%) (Table 2).

**Table 2:** **Listing of birth defects detected by timing of antiretroviral therapy exposure**

Birth defects	Earliest antiretroviral exposure		
	First trimester (n = 329)	Second/third trimester (n = 488)	None (n = 80)
**Central Nervous System**			
Microcephaly	0	1	0
**Face/neck/eyes/ears**			
Morrie syndrome	0	1	0
Microphthalmia	1	0	0
Coloboma	0	0	1
Syndromic facies	1§	0	0
**Cardiovascular and circulatory defects**			
Atrial septal defect	5	4	0
Ventricular septal defect	2	2	0
Transposition of the great arteries	1	0	0
Pulmonary valve stenosis	0	2*	0
Hypoplastic left heart syndrome	1†	0	0
**Respiratory**	0	0	0
**Digestive system**			
Hepatic venous malformation	0	1	0
Hepatic lymphangioma	0	1	0
Alagille syndrome	0	2#	0
Oesophageal atresia	1‡	0	0
Diaphragmatic hernia	1	0	0
**Genital organs**			
Cryptorchidism	3	1	2
Double urethra	0	1	0
Vaginal polyp	1	0	0
**Urinary system**			
Hydronephrosis	2	5	0
Hypospadias	0	3	0
Renal duplication and ureterocele	0	1	0
**Musculoskeletal**			
Premature synostosis	1	1	0
Clubfeet	0	1	0
Phocomelia	0	1	0
Congenital hip dislocation	3	0	0
Congenital knee dislocation	0	1	0
**Limbs**			
Syndatyly	0	1	0
Polydactyly	0	1	0
**Miscellaneous defects**			
Pierre Robin syndrome	0	1	0
Fetal alcohol syndrome	0	0	2
Trisomy 21	0	1	1
**Total infants with defects**	23	33	6

*Includes one case with atrial septal defect.

#Both patients also had tracheal stenosis.

†This patient also had hydronephrosis.

‡This patient also had vertebral bodies abnormalities and left superior caval vein.

§This patient also had blepharophimosis and developmental delay.

The risk of birth defects was not significantly associated with maternal age or ethnic origin. There were no differences either with smoking or using illicit drugs during pregnancy. When we analyzed immunological and virological factors no differences were found in BD prevalences associated with maternal CD4 lymphocytes count in first trimester or maternal viral load in third trimester (Table 3).

**Table 3:** **Risk factors for congenital abnormalities**

	Total		Congenital abnormality		P
	N	%	N	%	
**Maternal age at enrolment (n = 825)**					
<20 years	14	1.7	2	14.3	0.43
20-35 years	577	69.9	38	6.6	
≥35 years	234	28.4	19	8.1	
**Maternal ethnic origin (n = 897)**					
Caucasian	628	69.9	46	7.3	0.94
Latino American	96	10.7	6	6.3	
Sub Saharan	155	17.3	9	5.8	
Other	18	2.1	1	5.5	
**Smoking during pregnancy (n = 680)**					0.45
Yes	270	39.7	17	6.2	
No	410	60.3	33	8.0	
**Alcohol during pregnancy (n = 696)**					0.73
Yes	34	4.9	3	8.8	
No	662	95.1	49	7.4	
**Drugs (cocaine, heroin, methadone) (n = 750)**					0.78
Yes	74	9.9	6	8.1	
No	676	90.1	49	7.2	
**Maternal diabetes (n = 876)**					
Yes	51	5.8	6	11.8	0.16
No	825	94.2	56	6.8	
**Maternal CD4 lymphocytes in 1st trimester (n = 554)**					
<200 cells/μl	66	11.9	6	9.1	0.29
200-500 cells/μl	238	43.0	14	5.9	
>500 cells/μl	250	45.1	24	9.6	
**Maternal viral load in 3rd trimester (n = 642)**					
>50 copies/ml	173	26.9	16	9.2	0.92
<50 copies/ml	469	73.1	28	6.0	
**Treatment in first trimester (n = 897)**					1.00
Yes	329	36.7	23	7.0	
No	568	63.3	39	6.8	
**Mother’s treatment group (n = 818)**					0.24
Untreated	78	9.5	6	7.7	
Monotherapy	10	1.2	1	10.0	
Dual therapy	21	2.6	4	19.0	
HAART including PI	476	58.2	31	6.5	
HAART not including PI	233	28.5	14	6.0	
**Gestational age (weeks) (n = 850)**					0.07
>37	656	77.2	40	6.1	
<37	194	22.8	19	9.8	
**Newborn sex (n = 890)**					0.75
Male	471	52.9	34	7.2	
Female	419	47.1	28	6.7	
**Birth weight (n = 863)**					0.003
>2,500 g	642	74.4	35	5.4	
<2,500 g	221	25.6	25	11.3	

HAART, highly active antiretroviral treatment; PI, protease inhibitor.

Birth defects were associated with low birth weight in our cohort (11.3 vs 5.4%, p < 0.01) but although there was a slight difference, the association did not reach significance for preterm delivery (9.8 vs 6.1%, p = 0.07).

The type of treatment received by their mothers (none, monotherapy, dual therapy or HAART) was not associated with BD (Table 3).

In analysis there was no significant association with exposure to specific drugs. (Table 4). There were 42 infants exposed in utero to efavirenz; three of them, (7.1%) had birth defects. This percentage did not differ significantly from the prevalence of BD in infants without efavirenz exposure [7.1% (3/42) versus 7.0% (56/793); p = 1.00]. Only two children exposed to EFV in first trimester had BD (two hydronephrosis). No central nervous system anomalies were observed in infants exposed in utero to efavirenz. Two hundred eighty-seven children were exposed to ZDV in first trimester. Eighteen children exposed to ZDV in first trimester had BD (eight patients with birth defects in genital organs and urinary system and seven with birth defects in cardiovascular system). Two of the three cases of hypospadias were in infants exposed to zidovudine in utero. Fifty-nine children were exposed to ddI in first trimester and five of them were exposed to ddI in first trimester had BD (8.4%)

**Table 4:** **Prevalence of birth defects according to type of antiretroviral drug**

Antiretroviral drug during pregnancy	First trimester exposure	Second/third trimester exposure	No exposure	P ^a^	OR ^b^. First trimester exposure vs No exposure	aOR ^c^. First trimester exposure vs No exposure	OR ^b^. Second/third trimester exposure vs No exposure	OR ^c^. First trimester exposure vs Second/third trimester exposure
**NRTI**								
Zidovudine	18/287 (6.27%)	20/269 (7.43%)	19/289 (6.57%)	0.85	0.95 (0.48; 1.85)	1.21 (0.56;2.63)	1.14 (0.59; 2.18)	0.83 (0.43; 1.61)
Lamivudine	22/338 (6.51%)	17/285 (5.96%)	19/227 (8.37%)	0.54	0.76 (0.40; 1.44)	0.87 (0.42; 1.82)	0.69 (0.35; 1.36)	1.09 (0.57; 2.10)
Stavudine	7/96 (7.29%)*	3/48 (6.25%)	48/687 (6.99%)	0.97	1.04 (0.45; 2.38)	1.15 (0.49; 2.66)	0.88 (0.26; 2.96)	1.17 (0.29; 4.78)
Didanosine	5/59 (8.47%)*	5/38 (13.16%)	48/734 (6.54%)	0.27	1.32 (0.50; 3.46)	1.25 (0.42; 3.67)	2.16 (0.80; 5.79)	0.61 (0.16; 2.27)
Abacavir	4/73 (5.48%)*	3/18 (16.67%)	51/744 (6.85%)	0.24	0.78 (0.27; 2.24)	0.99 (0.34; 2.87)	2.71 (0.76; 9.69)	0.28 (0.05; 1.43)
Tenofovir	4/58 (6.90%)*	1/22 (4.55%)	54/755 (7.15%)	0.89	0.96 (0.33; 2.75)	1.01 (0.30; 3.42)	0.61 (0.08; 4.68)	1.55 (0.16; 14.73)
Emtricitabine	1/19 (5.26%)*	0/15 (0.00%)	58/807 (7.19%)	0.53	0.71 (0.09; 5.46)	NA	NA	NA
**NNRTI**								
Nevirapine	7/150 (4.67%)*	3/85 (3.53%)	47/611 (7.69%)	0.19	0.58 (0.26; 1.32)	0.53 (0.22; 1.30)	0.43 (0.13; 1.44)	1.33 (0.33; 5.31)
Efavirenz	2/30 (6.67%)*	1/12 (8.33%)	56/793 (7.06%)	0.98	0.94 (0.21; 4.04)	1.04 (0.23; 4.55)	1.19 (0.15; 9.43)	0.78 (0.06; 9.56)
**PI**								
Nelfinavir	11/127(8.66%)*	11/141 (7.80%)	36/562 (6.41%)	0.61	1.38 (0.68; 2.80)	1.56 (0.73; 3.34)	1.23 (0.61; 2.49)	1.12 (0.46; 2.68)
Saquinavir	1/40 (2.50%)*	1/28 (3.57%)	56/766 (7.31%)	0.39	0.32 (0.04; 2.41)	NA	0.46 (0.06; 3.52)	0.69 (0.04; 11.55)
Lopinavir/r	5/86 (5.81%)*	3/51 (5.88%)	51/696 (7.33%)	0.82	0.78 (0.30; 2.01)	0.88 (0.30; 2.55)	0.79 (0.23; 2.62)	0.98 (0.22; 4.31)
Indinavir	4/21 (19.05%)*	1/10 (10%)	54/810 (6.67%)	0.08	3.29 (0.92; 10.13)	3.74(0.94; 11.89)	1.55 (0.19; 12.50)	2.11 (0.20; 21.88)
Atazanavir	1/6 (16.67%)*	3/19 (15.79%)	50/780 (6.41%)	0.16	2.92 (0.33; 25.47)	3.27(0.68; 15.73)	2.73 (0.77; 9.70)	1.06 (0.08; 12.68)
Fosamprenavir	0/4 (0%)*	0/3 (0%)	59/837 (7.05%)	0.86	NA	NA	NA	NA
Tipranavir	0/1 (0%)*	0/0	59/843 (7.00%)	0.78	NA	NA	NA	NA
**Other ARVs**								
Enfuvirtide	0/0*	0/1 (0%)	59/846 (6.97%)	0.78	NA		NA	NA
Raltegravir	0/0*	0/1 (0%)	62/896 (6.92%)	0.79	NA		NA	NA

NRTI, nucleoside reverse transcriptase inhibitor; NNRTI nonnucleoside reverse transcriptase inhibitor; PI, protease inhibitor. NA not applicable/no child in this category.

*First trimester exposures less than 200 for each agent.

^a^P values are global p-value for the three categories in each type of exposure. P values were calculated using chi square test and Fisher’s exact test. ^b^OR obtained by univariate logistic regression. ^c^AOR obtained by multivariate logistic regression adjusted for age, alcohol and illegal drug use during pregnancy.

We observed a high prevalence of defects after atazanavir exposure, even when we broke down this result by trimester of exposure (16.7% in first trimester and 15.8% in second/third trimester). The defect after atazanavir exposure in first trimester was oesophageal atresia, and the defects after atazanavir exposure in second/third trimester were Pierre Robin syndrome, hydronephrosis and atrial septal defect.

As we observed differences between baseline characteristics of HIV infected mothers and time of earliest antiretroviral therapy during pregnancy, we analysed the association between birth defect and ARV treatment exposure adjusted for age, alcohol and illegal drug use, but not significant differences were found (Table 4).

## Discussion

A major birth defects prevalence of 6.9% was observed in this cohort. This population of infants born to HIV-infected women was similar to other European cohorts in aspects such as the characteristics of the mothers and rates of perinatal HIV transmission. However, the prematurity rates in our cohort are higher than in other cohorts [1],[5],[24]-[26]. The prevalence of BD in this cohort is higher than previously described. Knapp et al. compared different European [27],[28] and American studies [8],[13],[29] evaluating ARV exposure and congenital anomalies and observed a prevalence from 1.5 to 6.1% [9] Overall in most of these studies the physicians evaluated the infants for BD based on physical examinations during scheduled visits.

A difference has to be established between passive surveillance systems (most of which only recording defects based on physical examinations detected in scheduled visits) and active surveillance systems, which include ultrasound scans and echocardiogram in all infants with follow-up from birth. Active surveillance systems, usually show higher BD prevalences. In more than thirty thousand of German newborns not exposed to ART, an active vigilance study showed a BD prevalence of 6.9% [30].

We have reported 17 cardiac malformations, including 9 atrial septal defects and 4 ventricular septal defects. Some authors have reported that early screening echocardiography can detect important subclinical malformations and produce prevalences of cardiac defects of 5% to 10% higher than expected [31],[32]. We also reported two hepatic vascular anomalies and a renal duplicity, detected by ultrasonography. These BDs may not have been otherwise detected in a passive surveillance study. However we could not measure the impact of an active surveillance system used in our cohort.

There are obviously ethnic and environmental factors related to BDs. However we could not show any association between ethnic group and BD in our cohort (Table 3). In previous studies in Spanish general population in the last ten years, BDs prevalence was 2% based on two classification systems [33],[34]. Although these results differs considerably from the BD prevalence published in our study, they were not based on EUROCAT classification system. In these registries either systematic ultrasounds or long follow-up of the children were not included.

Preterm birth and birth defects, especially congenital heart defects have been previously associated. Based on these results, it has been suggested that an underlying mechanism or a genetic disorder could explain this association [35]. We could not report the association between preterm delivery and BD (9.8 vs 6.1%, p = 0.07), but we have observed an association with low weight at birth (11.3 vs 5.4%, p < 0.01). Interestingly, in our cohort 40% (25 cases) of BD were observed in patients with low weight at birth.

The use of illicit drugs during pregnancy (cocaine, heroine or methadone) was not associated with BD. Two cases of foetal alcoholic syndrome were documented. Mothers of these two children reported to consume large amounts of alcohol. However, we could not observe an association between alcohol consumption and BD. Alcohol was a self-reported variable, and it was an important limitation for its analysis.

We did not detect any significant association between BD prevalence and type or timing of ART in pregnancy. First-trimester antiretroviral exposure was not significantly associated with birth defects. This fact is consistent with most of the reports concerning BD and ART, which do not show an increased risk of teratogenesis in infants exposed to ART in the first trimester of pregnancy. However, others studies have found higher rates of BD associated a specific ARV drugs. Exposure to zidovudine in first trimester have been associated to higher prevalence of hypospadias [13] but also to heart defects [14]. In our study, we included more than 200 children with ZDV exposure in first trimester, and we didn’t observe an increased risk of BD. However in the French Perinatal Cohort with more than 3000 children exposed to ZDV in first trimester, a significant association was found with congenital heart defects.

According to the type of drug used (Table 4), no significant increase in BD prevalence was found for each drug, not even with EFV (6.67% in intrautero exposed infants vs 8.33% in no exposed infants; p = 0.98) neither ddI (8.47% vs 13.16%; p =0.27). In the case of atazanavir the rates were higher, but that because of the small number of women exposed, the confidence intervals were large and it was not possible to conclude.

Recently a significant association was found between indinavir and head and neck defects. In our cohort, we observed a higher prevalence of defects in children exposed to indinavir in the first trimester, however, this difference did not reach significance and did not concern head or neck defects.

This study has several limitations. In prospective cohorts the confounding factors are not sufficiently controlled and can lead to bias due to the interpretation of the results. We have not consider for analysis stillbirths and termination of pregnancy. Data as smoking or using illicit drugs during pregnancy were self reported. Also no other drugs used during pregnancy were analysed in this report. The major limitation of our study is the small number of children exposed to ARV in first trimester. We have not analysed any association of specific BDs with each ARV drug. For this reason, no conclusion could be assessed when the number of exposures in first trimester is low. Cohort collaborations and long term follow up are required to further analysis.

## Conclusion

In conclusion, a higher prevalence on BDs in infants born to HIV-infected women was observed in this report. However, in utero exposure to ART was not associated to BD. The relatively small number of patients is a major limitation for further analysis in this study.

## Appendix 1: Members of the Madrid Cohort of HIV-Infected Mother-Infant Pairs

Hospital Universitario 12 de Octubre: MI González-Tomé, E Muñoz, O Nieto, P Rojo, JM Hernández-García, R Rubio, F Pulido, D Lora, A Gómez de la Cámara, M de Matías, B Fraile, D Blázquez, LI González-Granado, A Navas.

Hospital Universitario La Paz: MI de José, JM Peña, M González, MJ Mellado, L Escosa.

Hospital General Universitario Gregorio Marañón: ML Navarro, J Saavedra, MD

Gurbindo, MC Viñuelas, P Miralles.

Hospital General de Móstoles: MA Roa, C Garaulet.

Hospital Severo Ochoa: I Olabarrieta, MJ Santos, N Martínez, G Alonso.

Hospital Príncipe de Asturias: J Beceiro, S Oñate.

Hospital Ramón y Cajal: A Holguín, P Rojas, C Fernández.

Hospital Universitario de Getafe: JT Ramos, B Soto, A Álvarez, M Fernández Ibieta, S Guillén, E Iglesias, I Cuadrado, I Solis, G Gaspar, L Prieto, I García de Diego.
